# Recovering Together: The Socioecological Impact of Social Networks on Postpartum Substance Use Disorder Recovery

**DOI:** 10.1007/s10597-025-01482-9

**Published:** 2025-06-21

**Authors:** Leah A. Holcomb, Caitlin Koob, Bonnie Treado, Rachel Mayo, Kathleen B. Cartmell, Jennifer Barkin, Lori Dickes, Kacey Eichelberger

**Affiliations:** 1 Department of Psychiatry and Behavioral Sciences, Medical University of South Carolina, Charleston, SC USA; 2 Department of Healthcare Leadership and Management, Medical University of South Carolina, Charleston, SC USA; 3 College of Health Professions, Anderson University, Anderson, SC USA; 4 Department of Public Health Sciences, Clemson University, Clemson, SC USA; 5 Department of Community Medicine and Obstetrics and Gynecology, Mercer University School of Medicine, Macon, GA USA; 6 Department of Political Science, Clemson University, Clemson, SC USA; 7 Department of Obstetrics and Gynecology, University of South Carolina School of Medicine Greenville, Prisma Health Upstate, Greenville, SC USA; 8 Women’s Reproductive and Behavioral Health, Medical University of South Carolina, 125 Doughty St., Suite 400, Charleston, SC USA

**Keywords:** Postpartum, Maternal functioning, Substance use disorder, Mental health

## Abstract

This study explored the experiences of postpartum individuals in early substance use disorder (SUD) recovery and key aspects of their social networks using the Socioecological Model (SEM). We conducted a qualitative study of 22 postpartum individuals (< 1 year since delivery) in residential SUD treatment from July to November 2023. Transcribed, audio-recorded interviews were analyzed using Atlas.ti, with findings organized by SEM levels. Participants, primarily White (81.8%), single (86.3%), and living below the poverty line (81.8%), reported opioid (40.1%) and stimulant (81.8%) use disorders. Challenges spanned SEM levels, including navigating parenthood, accessing trauma-informed care, and overcoming structural barriers such as housing instability, transportation, and childcare access. Findings underscore the need for family-centered, trauma-informed recovery programs and flexible policies addressing childcare and social service barriers to support long-term recovery and maternal well-being.

**Supplementary Information:**

The online version contains supplementary material available at 10.1007/s10597-025-01482-9.

## Introduction

Substance use during the perinatal period—spanning from pregnancy through the first year postpartum—is a growing public health concern with significant implications for maternal and infant outcomes in the United States. In the US, recent data indicate a notable rise in perinatal substance use, with an estimated 2.7% of pregnant individuals diagnosed with a substance use disorder (SUD) (Frankeberger et al., 2023). The postpartum period, in particular, is a uniquely complex and challenging time for individuals, marked by significant physical, emotional, and social transitions (Wszolek et al., 2019). For those with SUD, this complexity is further heightened by the ongoing demands of recovery, co-occurring mental health challenges, and the heightened risk of relapse or overdose, particularly between seven and twelve months postpartum (Glazer & Howell, 2021; Kountanis et al., 2023; White et al., 2023a, b). Even individuals without an SUD often experience considerable strain during this time, including physical recovery from childbirth, hormonal changes, sleep deprivation, and the demands of caring for a newborn (Wszolek et al., 2019).

Navigating the postpartum period is particularly difficult for individuals who lack a robust social support network, which is critical for emotional well-being, practical assistance, and the mitigation of stressors that can impede overall functioning (Barkin et al., 2014, Reid & Taylor, 2015). For those with SUD, these challenges are often compounded by stigma, fear of punitive action, and barriers to accessing treatment, leaving many without adequate support (Kotelchuck et al., 2017; Nielsen et al., 2020). A strong social support network—encompassing family, friends, peers, and healthcare providers—can be a crucial protective factor during this time, offering emotional reinforcement, facilitating access to care, and reducing feelings of isolation (Islam et al., 2023, Brown et al., 2013). This has left a critical gap in understanding how to support postpartum individuals with SUD, especially during the months when maternal mental health crises and drug-related deaths are most prevalent. The present study directly addresses this gap by examining how postpartum social support networks influence recovery experiences among individuals with SUD. By focusing specifically on the postpartum period, this research aims to inform the development of more targeted, sustained, and supportive interventions that extend beyond pregnancy into the high-risk postpartum phase. Social support is a central component of overall functioning in the postpartum period and plays a critical role in promoting recovery and reducing relapse risk for individuals with SUDs (Islam et al., 2023, White et al., 2023a, b). While perceived social support measures provide valuable insights, it is equally important to contextualize this support within the broader network of relationships and resources available. The socioecological model (SEM) offers an effective framework for examining these complex social dynamics, considering the interplay between individual, interpersonal, community, and societal factors (Bronfenbrenner, 1977). Based on the idea that individuals are both affected by and affect a complex range of social influences and environmental interactions, SEM highlights how factors like social networks, stigma, and systemic barriers shape the recovery process. These influences can cross multiple levels and impact people differently based on their cumulative life experiences. Loneliness is particularly pervasive during the postpartum period, a time marked by significant physical, emotional, and social transitions, which can exacerbate feelings of isolation and create additional challenges for individuals navigating recovery from SUDs (Adlington et al., 2023). For postpartum individuals with SUDs, the SEM framework offers a lens to examine both personal recovery efforts and the environmental and relational factors that can either support or hinder sustained recovery and effective parenting. This study explores the role of social networks and systems in shaping SUD recovery experiences and outcomes, using the SEM to identify key components of social support and needs through qualitative interviews with postpartum individuals in early SUD recovery.

## Materials and Methods

### Study Design and Data Collection

To explore the experiences of postpartum individuals navigating motherhood and SUD recovery, we conducted individual interviews designed to foster privacy and encourage open discussion of sensitive topics (Heath, et al., 2018). The interview guide was developed through a rigorous two-phase refinement process. Initially, it was tested with a small group of experts and patient participants (*n* = 5) to evaluate its clarity, cultural relevance, sensitivity, and establish content validity. Feedback from this phase prompted adjustments, such as adding contextually relevant prompts and adopting a language style more conducive to a conversational tone to enhance participant engagement. Subsequently, a multidisciplinary team of experts in the clinical content and qualitative methodology reviewed the revised guide to ensure it aligned with best practices in qualitative research. The finalized version included 20 core questions, supplemented by 16 follow-up prompts to generate nuanced and detailed responses.

This study received approval from the Clemson University Institutional Review Board. Participants were purposively recruited from a residential treatment facility in the Southeastern United States, chosen for its unique organizational structure that allows mothers to undergo treatment while residing with and caring for their children (aged < 6 years). Eligible participants were English-speaking, gave birth within the past year, and were diagnosed with an SUD within the past five years. Recruitment involved verbal invitations from study team members, who also provided a non-signed consent form detailing the study’s purpose and procedures. Participants were recruited from a larger study examining maternal functioning (Holcomb et al., 2024) within the context of SUD recovery. Interviews last 20 to 40 min and were conducted in person at the treatment facility between July and November 2023. With participant consent, all sessions were audio-recorded to ensure accurate data capture and were transcribed verbatim. A brief demographic survey, completed before the interview, provided additional context. Participants received a $35 gift card to acknowledge their time and insights.

### Data Analysis

Audio recordings were transcribed and de-identified by a professional transcription service. To ensure accuracy, all transcriptions were cross-checked against the original recordings and edited as needed. The interview data were analyzed using a thematic analysis approach to generate primary and sub-themes. Two members of the study team independently coded the transcripts, using predefined deductive codes based on the SEM and generating additional inductive codes related to each SEM domain. These subthemes were then grouped under the relevant domains of the SEM (individual, interpersonal, institutional/organizational, community, policy) to better understand the broader contextual factors influencing participants'experiences. Midway through the coding process, the coders met to compare their findings and reach consensus on the themes, refining the codebook as necessary. After finalizing the codebook, all transcripts were re-coded to ensure consistency and accuracy. Atlas.ti version 25.0 and SAS version 9.4 software were used to analyze the data.

## Results

### Sample Characteristics

A total of 22 postpartum women in recovery participated in the study. Participants were predominately White (81.8%), single (86.3%), and between 20 and 40 years of age. All participants had at least some high school education, an annual household income of less than $15,000 a year (81.8%) and were the primary caregivers for their children (63.6%). Roughly half of the participants were less than three months postpartum (59.1%), and almost all (90.9%) had experienced at least two pregnancies. Participant characteristics are shown in Table 1. Most participants reported having either an opioid use disorder (40.9%) or stimulant use disorder (81.8%), or polysubstance use disorder (40.9%).

**Table 1:** Characteristics of Postpartum People in Recovery (*n* = 22)

Characteristic	% ( *N* )
*Age*	
20–29	54.6 (12)
30–39	36.4 (8)
40 +	9.1 (2)
*Race*	
White	81.8 (18)
Black/African American	9.1 (2)
Other	9.1 (2)
*Ethnicity*	
Not Hispanic/Latino	90.9 (20)
Hispanic/Latino	9.1 (2)
*Marital Status*	
Single	86.3 (19)
Married	4.6 (1)
Separated	4.6 (1)
Divorced	4.6 (1)
*Education Level*	
Some high school	36.4 (8)
Highschool diploma or GED	36.4 (8)
Some college	27.3 (6)
*Annual Household Income Level*	
Less than $15,000	81.8 (18)
Greater than $15,000–24,999	9.1 (2)
Prefer not to answer	9.1 (2)
*Gravida*	
1	9.1 (2)
2	18.2 (4)
3	31.8 (7)
4	27.3 (6)
5	13.6 (3)
*Age of youngest child*	
0–3 months	59.1 (13)
4–6 months	9.1 (2)
7–11 months	9.1 (2)
12 + months	22.7 (5)
Primary Childcare Mode*	
Care for child 100% of the time	63.6 (14)
Share childcare with a spouse/partner	9.1 (2)
Share care with a family member	18.2 (4)
Child attends daycare	27.3 (6)
SUD Type*	
Polysubstance Use Disorder	40.9 (9)
Opioid Use Disorder	40.9 (9)
Stimulant Use Disorder	81.8 (18)
Cannabis Use Disorder	13.6 (3)
Alcohol Use Disorder	4.6 (1)
Sedative Use Disorder	9.1 (2)

Table 1 provides an overview of participant characteristics, reported as frequencies

*Percentages may exceed 100% since participants could report multiple SUDs

### Primary Themes

**Fig. 1:**
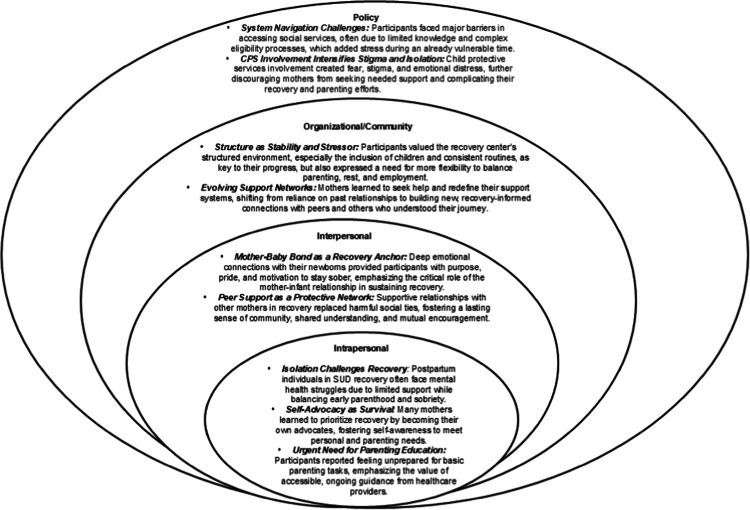
Primary findings mapped onto the SEM framework

Primary sub-themes are organized according to the five levels of the SEM. Table 2 provides complementary quotes for each reported theme. We identified ten sub-themes related to social networks and substance use disorder recovery during the postpartum period: within the individual level, 1) *Navigating Early Parenthood in Isolation While Sustaining Recovery* and 2) *Seeking Guidance and Support Through Parenting Education;* within the interpersonal level, 3) *Developing Deep and Distinctive Connections with Their Newborn* and 4) *Building Supportive Peer Relationships to Replace Unsupportive Friends and Partners*; within the institutional/organizational level, the 5) *The Role of Recovery Center Structure in Shaping the Recovery Experience* and 6) *Learning to Seek Help and Redefining Support Networks in Recovery*; within the community level, 7) *Respectful, Trauma-Informed Prenatal Care as a Catalyst for Recovery Engagement,* 8) *Impact of Housing and Transportation Insecurity on Recovery and Parenting,* and 9) *Access to Childcare as a Critical Support for Recovery and Parenting*; within the policy level, 10) *Barriers to Accessing Social Services and the Impact on Recovery and Parenting.* Figure 1 provides a visual representation of the study’s results fitted to the SEM framework.

**Table 2:** Themes and Relevant Quotes (*n* = 22)

Theme	Direct Quote
*Individual Level*	
Navigating Early Parenthood in Isolation While Sustaining Recovery	*I went through my pregnancy completely alone…my mental health took a really bad hit in my first trimester, and then I got sick in the third, so I was on six different medications. And then, I was – then I started using drugs and it was a bad decision. So, when I first had him I felt empty…I looked at [my baby] and I had love, I could feel love where I knew I loved him but I couldn't feel love. I couldn't feel joy. I felt just empty, just really empty*
	*Mental health is a big deal. It's something that I never really balanced before. As a mom, I was just about working or taking care of my kids, or focusing on physical health, but mental health was not something that I was really prioritizing in my life. It's something that I think is probably the most important thing*
	*I had this idea of a perfect pregnancy, perfect family thing. I didn't want to have a baby in a broken home or in a home where I – it wasn't even that – I didn't want to have a baby without being able to care for him the way that they deserve*
Seeking Guidance and Support Through Parenting Education	*But that was on me. I should have just put him right back in the bed. But he's got to the point now where I can put him in the bed after I feed him, and he doesn't cry. If he does, it's just for a few minutes…whenever he was smaller, he would get upset and want to be held. And I always thought I had to hold him until he fell back asleep. But it's better not to do that, because he transfers better, just putting him in there*
	*One thing is no one gave me a manual and it’s a learning experience every day, every day. I’m learning something new every day on how to be a better mama*
*Interpersonal Level*	
Developing Deep and Distinctive Connections with Their Newborn	*She gave me a purpose. My other children were taken away for drugs and I just wouldn't have it this time because I just couldn't stop using when I was pregnant. But this pregnancy, I didn't touch period with her at all. And I just won't lose her for no reason*
	*She helps with that because I know I can’t use with her or I’ll lose her and I’m not losing her. So that’s how she helps with that. She’s my reason*
Building Supportive Peer Relationships to Replace Unsupportive Friends and Partners	*It's a whole lot harder to do, just being newly sober… I wouldn't really say I wish I would have known anything, because I'm like learning everything. But I have a mom that'll support me and help me out if I need it, and my friends here*
	*My friend, I've known her outside of here before, and we've gotten like super close since she came here. And we've been helping each other out. And she helps me a lot. I help her a lot, recovery-wise and kid-wise*
	*Now that I'm in recovery, I feel like everybody's supporting me. I have people here. I have people at the meetings*
*Institutional/Organizational Level*	
The Role of Recovery Center Structure in Shaping the Recovery Experience	*You know, here, any meetings, classes, you know, we can take the baby…But I don't know if it may in the future or not. But it's working out pretty good right now."*
	*Going to meetings. Finding a network of strong women that are around me, hold me accountable…But just that – having a strong network of strong women – that's helpful, you know?*
	*Definitely when I was in the rehab part, I definitely felt like that big time, like we were pretty much – we were told it's mandatory to do this and other stuff could wait until later, and sometimes later never came. We didn't really have a whole bunch of downtime at all, or time with our children, I felt like. That really hurt a lot. I feel like over here, I'm getting to balance more of that and try and make up for all of that time with my child that I lacked over there, and actively putting more work in on myself*
Learning to Seek Help and Redefining Support Networks in Recovery	*Well, I’ve received support from my sons. Like one bought her a pack and play. And then he would come and ask if he could be on visits. But he’ll come to see me here and there and make sure I’m coping well*
	*Ask for help. It's okay to ask for help. It's not going to make you any lesser of a mother*
	*I know that if I need to be like,"Well, can you watch the baby for a few minutes just so I can take a shower or take a nap or whatever?"I know that I do have people here that'll help me when it comes down to that situation. But I used to be real – what do you call – like, too – my pride was too big to ask for help. But now, I know that it's nothing wrong with asking for help when you need it*
*Community Level*	
Respectful, Trauma-Informed Prenatal Care as a Catalyst for Recovery Engagement	*The support I got at the hospital from the Magdalene Clinic, things like that – it's just top tier support*
Impact of Housing and Transportation Insecurity on Recovery and Parenting	*[I could use] more help with resources, finding resources, like diapers and clothes, and just necessary household items and necessary things that I need for the baby when he comes home*
	*The Medicaid I have pays for ModivCare. And that helps provide transportation for us to our doctor's appointments and back. And so even though I may arrive like an hour early, we still get there and get back*
	*I can use the Medicaid van for transportation – which I do have to set up for my appointments and to go to the methadone clinic. But there's another girl in the back that goes to the clinic so, she lets me ride with her. And so, that's a big help so that I don't have to ride the Medicaid van'cause sometimes, you have to wait an hour for them to come back and get you*
Access to Childcare as a Critical Support for Recovery and Parenting	*Trying to find childcare, a daycare for her to go to and [in] a reasonable price range. And they’re so backed up, like got a long waiting list*
	*That’s a really huge obstacle, I guess. Because sometimes I want to put her in daycare and I can’t get there, let’s say in the bus*
*Policy Level*	
Barriers to Accessing Social Services and the Impact on Recovery and Parenting	*The [baby’s] father doesn't want to be involved but I can't get TANF and stuff like that without risking him finding out because DSS will inform him*
	*Well, this place provides us with ABC vouchers, so. But I applied for WIC and food stamps, and we've got both of them started. So really, I mean, I buy – like WIC pays for his food. But because I was breastfeeding, and as I was gradually going up on it, I couldn't give him what he needed…So I use what the hospital told me, Similac, the closest thing to breastfeeding, and I just started buying that with the food stamps for him. And he seems to be doing quite well*
	*You gotta use your food stamps and I don't get food stamps until after I get the baby's social security number and stuff'cause I got a felony drug charge so, I can't get food stamps. But I do get WIC, but I do have to add him to both of those. But I guess I gotta wait on the birth certificate and a social security card before I can do that*

#### Individual Level

### Navigating Early Parenthood in Isolation While Sustaining Recovery

Navigating early parenthood while recovering from SUD during the postpartum period can be particularly challenging without a strong support system. Many participants described the significant toll this lack of support took on their mental health, as they struggled to balance the demands of motherhood with the challenges of maintaining sobriety. Some participants emphasized the importance of learning to be their own best supporter and advocate, recognizing the need to prioritize their recovery above all else. This shift involved developing greater self-awareness of their ongoing needs, which allowed them to focus on their well-being and make informed decisions that supported both their recovery and their role as a mother.

### Seeking Guidance and Support Through Parenting Education

Many participants expressed a lack of understanding when it came to fundamental parenting tasks, such as safe sleep practices, feeding routines, and approaches to discipline. This gap in knowledge left them feeling unprepared for motherhood and eager for more education from healthcare providers. Even mothers with older children noted that this was often the first time they learned essential concepts related to infant and child development, highlighting a critical need for consistent and accessible parenting education throughout the postpartum period.

#### Interpersonal Level

### Developing Deep and Distinctive Connections with Their Newborn

Many participants described forming deep bonds with their new babies, often calling their child their primary motivation for staying sober. The love and responsibility they felt provided a sense of purpose and direction, reminding them of what they stood to lose if they relapsed. These connections gave participants strength, pride, and determination, which highlights the critical role of the mother-baby dyad and its unique bonding experience in supporting recovery.

### Building Supportive Peer Relationships to Replace Unsupportive Friends and Partners

Having other mothers navigating similar challenges provided a critical source of support, often replacing relationships with friends or family members who were still using substances or unsupportive of their recovery. This shared experience fostered a sense of community described as"co-parenting"or forming a"village,"where participants could rely on one another for practical advice, emotional encouragement, and mutual understanding. The relationships formed within the program were viewed as enduring sources of support, with participants anticipating that these bonds would continue to facilitate their recovery and parenting trajectories beyond the duration of the program.

#### Institutional/Organizational Level

### The Role of Recovery Center Structure in Shaping the Recovery Experience

Mothers emphasized the importance of being able to include their children in programming and bring them to classes, describing it as vital to their ability to stay in recovery. Many noted that the structure provided by the center played a critical role in supporting their recovery, as for some, this was the first time they had experienced a consistent daily schedule. This structure offered a new learning opportunity, helping them establish routines and manage their time effectively. However, some mothers also expressed feeling overwhelmed by the constant demands of structured meetings and classwork. They voiced a desire for more time to bond with their children, pursue employment opportunities, and receive additional support for nighttime caregiving. These reflections highlight the dual importance of structure and flexibility, as well as the need for wraparound services that balance recovery programming with the practical and emotional demands of parenting.

### Learning to Seek Help and Redefining Support Networks in Recovery

Many mothers described how they had to learn to ask for help, a skill they had rarely utilized before entering the program. This process of reaching out for support was a significant shift, as many had previously struggled with relying on others. In addition to learning how to ask for help, they also realized that their support networks needed to evolve. While they had relied on family and partners in the past, they found that their recovery journey often led them to build new relationships and seek support from peers and others who understood the unique challenges they were facing.

#### Community Level

### Respectful, Trauma-Informed Prenatal Care as a Catalyst for Recovery Engagement

Participants who accessed a partnering trauma-informed prenatal care provider consistently emphasized the importance of being treated with respect and without judgment. Many described the positive impact of having care providers who approached them with empathy and understanding, rather than stigmatizing attitudes. Participants reported that such care not only made them feel more supported but also helped improve their engagement in treatment, reducing the barriers created by stigma.

### Impact of Housing and Transportation Insecurity on Recovery and Parenting

Participants highlighted the challenges of securing stable housing and reliable transportation after discharge, noting that these barriers hindered their ability to maintain recovery and meet parenting responsibilities. Many expressed that the lack of stable housing created instability, while transportation issues made it difficult to attend appointments, access resources, or find employment.

### Access to Childcare as a Critical Support for Recovery and Parenting

Participants described navigating daycare enrollment as a significant challenge, often relying on family for childcare. However, many noted that the recovery center's provision of on-site daycare was a crucial support, particularly for those transitioning from intensive residential care to more independent living. This service had a substantial impact, allowing mothers to focus on their recovery and parenting responsibilities without the added stress of securing childcare. For many, access to daycare facilitated their ability to engage in programming and work towards greater independence, significantly supporting both their recovery and their role as a mother.

#### Policy Level

### Barriers to Accessing Social Services and the Impact on Recovery and Parenting

Participants reported significant challenges in accessing social services, including obtaining Medicaid and food assistance programs (such as the Special Supplemental Nutrition Program for Women, Infants, and Children), often citing a lack of knowledge about available resources. Navigating these systems was a frequent source of frustration, as many struggled to understand eligibility criteria and the process of securing assistance. Additionally, complications related to child protective service (CPS) involvement further hindered their ability to access necessary resources for themselves and their children, creating additional layers of stress and anxiety. Many participants reported feeling stigmatized and isolated due to the scrutiny and intervention of CPS, which not only complicated their ability to navigate essential systems but also created a fear of judgment that impacted their willingness to seek help.

## Discussion

These findings highlight the complex social and ecological environment that postpartum people with SUDs experience during recovery, highlighting the importance of addressing individual, interpersonal, community, and policy level factors. These insights, modeled utilizing the SEM, reflect the interplay of personal and systemic influences that shape SUD recovery in the postpartum period. Use of a systems level perspective is particularly critical during major, transitions from residential to outpatient care—a vulnerable period characterized by gaps in continuity of care and fragmented systems (Manuel et al., 2017).

At the individual level, participants’ narratives reflected the unique pressures of early parenthood, including the mental and emotional toll of balancing recovery with the demands of motherhood. Many participants recognized their newborns as their primary motivation for recovery, a finding aligned with the concept of modified codependency, where attachment to the child becomes a central driver for sobriety (Lander et al., 2013). However, this reliance on external motivators may present risks if recovery efforts are not supported by personal resilience or a robust social support system (Manuel et al., 2017). Parenting education emerged as a critical need, particularly for mothers with limited prior exposure to child development knowledge or safe caregiving practices. Improving ongoing educational resources for this population are critical for improving parent–child relationships as well as early childhood outcomes (Arria et al., 2013). Additionally, the impact of trauma on participants’ ability to manage recovery highlights the need for trauma-informed interventions that build emotional regulation skills and support sound parenting decisions.

Within the interpersonal level, relationships played a pivotal role in recovery trajectories. The mother-baby dyad approach to care surfaced as a cornerstone of participants’ motivation, with the bond to their child serving as both a protective factor and a potential source of stress. The literature reinforces pregnancy and postpartum periods as critical intervention points for SUD, emphasizing the transformative potential of these life stages (Martin & Parlier-Ahmad, 2021). Peer-based models of support also emerged as historically effective for fostering long-term recovery (Fallin-Bennett et al., 2020). Participants described peer relationships as indispensable, offering mutual understanding, practical advice, and emotional encouragement. These findings validate the importance of embedding peer-support components into recovery programming, particularly for individuals who are simultaneously navigating new motherhood and SUD recovery.

At the institutional level, participants’ mixed feelings about recovery facilities highlight the need for interventions that balance structured support with individual flexibility. Future programs should assess participant readiness and tailor approaches to accommodate varying levels of engagement, ensuring that structured programming does not inadvertently become a barrier to sustained recovery (Raynor et al., 2017). Integrating adaptable scheduling, childcare options, and employment support within recovery services may enhance accessibility and long-term success for postpartum individuals navigating life while balancing the dual demands of parenting and sobriety (Coe et al., 2024).

From a community perspective, a destigmatizing, trauma-informed approach to prenatal care reported by participants played a pivotal role in fostering engagement and reducing barriers to treatment, underscoring its importance in supporting individuals with SUD throughout their recovery journey (Morton Ninomiya et al., 2023). This emphasis on trauma-informed care underscores the critical role of empathetic, nonjudgmental providers in fostering trust and engagement (Guille et al., 2022). Continuing this respectful, empathetic care model into the postpartum period is essential for sustaining recovery, as it reinforces a supportive environment that empowers individuals to maintain their recovery while fulfilling their parenting responsibilities. Future interventions should prioritize coordinated, cross-sector partnerships to address structural barriers like housing instability, transportation challenges, and childcare needs.

Finally, policy-level challenges underscore the critical need for targeted interventions to address systemic barriers faced by postpartum individuals in recovery, particularly single parents, who made up the vast majority (86.3%) of study participants. These individuals often face heightened burdens due to the dual responsibilities of caregiving and recovery, compounded by limited access to emotional, financial, and logistical support (Kim & Kim, 2020). Navigating fragmented social service systems, such as Medicaid and nutritional assistance programs, not only adds stress but also exacerbates disparities in access to essential resources (Grand-Guillaume-Perrenoud, et al., 2022). To mitigate these challenges, future interventions should prioritize simplifying enrollment processes, expanding eligibility criteria for single parents, and integrating case management services to streamline access to benefits (Hudon et al., 2022). Childcare needs, consistently highlighted as a significant barrier, call for innovative policy solutions, such as subsidized daycare programs tailored to mothers in recovery, legal protections for parenting individuals in treatment, or expanding on-site childcare services in recovery facilities (Gaur et al., 2024). In our sample, 81.8% of participants reported an annual household income of less than $15,000, highlighting that recovery efforts or employment opportunities are unlikely to be sustainable without access to subsidized or free childcare. Strengthening legal protections against discrimination in employment, housing, and child custody for single mothers in recovery is also essential to promoting equity and stability. Addressing these gaps can enhance maternal and child health outcomes by reducing stressors, fostering engagement in recovery programs, and supporting sustained recovery through holistic, family-centered care models (Bosak et al., 2024).

### Limitations

Findings of this research should be considered with several limitations in mind. Participants were predominantly White and reported using opioids or methamphetamines, which may limit the generalizability of findings to other racial groups or individuals who use other substances. Additionally, all participants were recruited from a single recovery center that allowed them to continue caring for their young children, which may not reflect the experiences of postpartum individuals navigating recovery without their children or those who received care at a different facility or those only receiving outpatient treatment. Additionally, many of the participants described receiving trauma-informed perinatal care, and as such their views might not be reflective of postpartum people who were unable to access trauma-informed perinatal care. Variations in interview length could also have constrained the exploration of more complex aspects of functioning, potentially overlooking nuanced experiences among some participants.

## Conclusions

This study explored the experiences of postpartum individuals in early SUD recovery, using the SEM to guide our examination of how individual, relational, and systemic factors shape recovery trajectories. Through in-depth qualitative interviews with 22 postpartum individuals in residential treatment, the analysis revealed intersecting challenges related to parenting while in recovery, lack of trauma-informed care, and persistent structural barriers such as housing instability, childcare inaccessibility, and fragmented social services. Despite these challenges, participants also identified sources of resilience and support, including peer relationships, family connections, and trusted providers. These findings underscore the urgent need for trauma-informed, family-centered recovery programs that recognize the complexity of postpartum recovery and integrate peer support, wraparound services, and targeted interventions addressing unmet social determinants of health. Critically, these findings highlight the disproportionate burden faced by single mothers living in poverty and emphasize the need for flexible policy solutions that expand access to childcare, simplify benefit navigation, and strengthen protections for parenting individuals with SUD. By adopting a system-level perspective and embedding support across all levels of the socioecological model, future interventions and policies can more effectively promote sustained recovery, maternal functioning, and the long-term well-being of mother–baby dyads. These insights can inform both clinical practice and policy reform, helping to close persistent gaps in postpartum SUD care and improve outcomes for this high-risk population.

## Supplementary Information

Below is the link to the electronic supplementary material.

Supplementary file1 (DOCX 19 KB)
