# Supplementary material for: Recovering Together: The Socioecological Impact of Social Networks on Postpartum Substance Use Disorder Recovery
Source: Community Ment Health J. 2025 Jun 21;61(8):1471–80. doi: 10.1007/s10597-025-01482-9 (PMC12647300; doi:10.1007/s10597-025-01482-9)
Supplement: Supplementary file 1 — Supplementary file1 (DOCX 19 KB) [file 10597_2025_1482_MOESM1_ESM.docx]

Supplemental File 1

Moderator Guide for Interviews with Postpartum People

*Hello, thank you for joining me today. My name is _____, and I am a graduate student at [redacted]. How are you today? *pause* Thank you for your time and your interest in this study. Before we begin, I would like to make sure that you qualify for the study. 1) Did you recently give birth? 2) Have you been diagnosed previously with a substance use disorder (SUD)?*

*AFTER MOTHER ANSWERS: Great, before we start, where would you feel most comfortable talking with me (outside, inside, etc.)? I want to tell you a little bit about the study. I am a Ph.D. student at [redacted]. I am trying to learn how moms care for themselves after having a baby. The information you provide will be used to help create clinical programs to support women with SUDs after having a baby. Do you have any questions for me?*

*Our discussion will last about 20 to 40 minutes to respect your time, but there is no time limit. Before we start, I would like to remind you that there are no right or wrong answers in this discussion. I am interested in knowing how you are doing after giving birth to your baby. I won’t keep any personal information that could identify you as part of our discussions.*

*Please feel free to stop me and ask questions at any time. Also, since we will be speaking for a while today, and it’s hard for me to remember everything, would it be okay if I recorded our conversation?*Pause**

*Thank you! I will write out your responses, ensuring confidentiality, and the recording will be destroyed after the files are transcribed. I will begin recording now.*

*Introduction/Ice Breaker:*

1. So, tell me a little bit about your baby.

*Management/Adjustment*

1. Having a baby is a new experience for most people. Can you tell me what it’s been like for you these last few weeks (or months) since you had your baby?
   1. Probe: How has having the baby changed your everyday life?
   2. Probe: How are you adjusting to these changes?
2. What new responsibilities have you taken on since having your baby?
3. Being a new mom can is challenging. What have been some of the biggest challenges you’ve experienced?
   1. Probe: What have been some of the biggest joys? *(If mom doesn’t answer, mention that it is okay to not have any right now and that she is not alone in her experience.)*
4. What support could have helped prepare you for having your baby?

*Infant Care/Mother-Child Interaction*

1. How much time do you spend with your baby each day?
2. What things affect the time you can spend with your baby?
3. How comfortable are you taking care of your baby’s needs?
4. What things about taking care of your baby do you think have been most difficult?
5. What do you wish you would have known about taking care of your baby before delivery?
   1. Probe, what about after having your baby?

*Psychological Well-being*

1. How have you been feeling emotionally since having your baby?
   1. Probe: Have you felt sad, lonely, or anxious?
   2. Probe: Could you describe some specific things that make these feelings you’ve described worse?
   3. Probe: Do you like to do anything to make yourself feel better?
   4. Probe: Going for a walk, reading, art?

*Self-Care*

1. We often describe self-care as someone taking an active role in managing their health. How would you describe your self-care since having a baby? *(This could be something like getting your nails done, going for a walk; or essentially anything you do to take some time for yourself.)*
   1. Probe: How have you tried to take care of yourself since having your baby?
   2. Probe: What things do you feel are important when taking care of yourself?
   3. Probe: What are things you want to do to practice self-care but have been unable to do since having a baby?
   4. Probe: If you could do something ‘fun,’ what would you do?”
2. Do you ever feel that taking care of your SUD gets in the way of taking care of your baby? If so, how?
   1. Probe: What are things that you do to care for yourself to try to keep from using?
3. Have your strategies to manage your substance use disorder changed since having a baby? Can you tell me about them?

*Social Support*

1. What kind of support from others have you received since having your baby?
   1. Probe: What kind of support do you feel you need and have not gotten?
2. Who has been your biggest supporter since having your baby?
   1. Probes: Partner, Spouse, parent, friend, provider?
   2. Probe: Could you describe things they have done to help support you?
3. What sort of support did you receive for handling your SUD since having your baby (from your provider, from the counselor, from a friend)?
4. Do you ever feel lonely as a new mom? (i.e. no one understands the difficulties you go through managing your SUD PP) If so, please describe.
5. What types of support do you wish you could have received to help you manage your health, specifically related to your SUD, since delivering your baby)?
6. What advice would you give to someone else with a similar experience of having a SUD and being a new mother? **what would you have wanted to know**

*CLOSING: Do you have anything else you would like to add? Do you have any questions for me? Thank you very much for your time today. *Have patient sign for gift card on tracking form**
